# Novel Synthesized *N*-Ethyl-Piperazinyl-Amides of C2-Substituted Oleanonic and Ursonic Acids Exhibit Cytotoxic Effects through Apoptotic Cell Death Regulation

**DOI:** 10.3390/ijms222010967

**Published:** 2021-10-11

**Authors:** Oxana Kazakova, Alexandra Mioc, Irina Smirnova, Irina Baikova, Adrian Voicu, Lavinia Vlaia, Ioana Macașoi, Marius Mioc, George Drăghici, Ştefana Avram, Cristina Dehelean, Codruța Şoica

**Affiliations:** 1 Ufa Institute of Chemistry, UFRC RAS, 71, pr. Oktyabrya, 450054 Ufa, Russia; si8081@yandex.ru (I.S.); poljr@anrb.ru (I.B.); 2 Faculty of Pharmacy, “Victor Babes” University of Medicine and Pharmacy, 2nd Eftimie Murgu Sq., 300041 Timisoara, Romania; alexandra.petrus@umft.ro (A.M.); laviniav@umft.ro (L.V.); macasoi.ioana@umft.ro (I.M.); marius.mioc@umft.ro (M.M.); draghici.george.andrei@gmail.com (G.D.); stefana.avram@umft.ro (Ş.A.); cadehelean@umft.ro (C.D.); codrutasoica@umft.ro (C.Ş.); 3 Research Center for Pharmacotoxicological Evaluations, Faculty of Pharmacy, “Victor Babes” University of Medicine and Pharmacy Timisoara, 2nd Eftimie Murgu Sq., 300041 Timisoara, Romania; 4 Faculty of Medicine, “Victor Babes” University of Medicine and Pharmacy, 2nd Eftimie Murgu Sq., 300041 Timisoara, Romania; 5 Formulation and Technology of Drugs Research Center, ”Victor Babeș” University of Medicine and Pharmacy, Faculty of Pharmacy, Eftimie Murgu Sq. No. 2, 300041 Timișoara, Romania

**Keywords:** oleanonic acid, ursonic acid, arylidene, piperazine, anticancer activity, NCI-60, cytotoxic activity, CAM assay, rtPCR, molecular docking

## Abstract

A series of novel hybrid chalcone N-ethyl-piperazinyl amide derivatives of oleanonic and ursonic acids were synthesized, and their cytotoxic potential was evaluated in vitro against the NCI-60 cancer cell line panel. Compounds **4** and **6** exhibited the highest overall anticancer activity, with GI_50_ values in some cases reaching nanomolar values. Thus, the two compounds were further assessed in detail in order to identify a possible apoptosis- and antiangiogenic-based mechanism of action induced by the assessed compounds. DAPI staining revealed that both compounds induced nuclei condensation and overall cell morphological changes consistent with apoptotic cell death. rtPCR analysis showed that up-regulation of pro-apoptotic Bak gene combined with the down-regulation of the pro-survival Bcl-XL and Bcl-2 genes caused altered ratios between the pro-apoptotic and anti-apoptotic proteins’ levels, leading to overall induced apoptosis. Molecular docking analysis revealed that both compounds exhibited high scores for Bcl-XL inhibition, suggesting that compounds may induce apoptotic cell death through targeted anti-apoptotic protein inhibition, as well. Ex vivo determinations showed that both compounds did not significantly alter the angiogenesis process on the tested cell lines.

## 1. Introduction

Malignant diseases have always been present in the history of mankind; in fact, the name “cancer” is related to the Greek physician Hippocrates (460–370 BC), who used the term “carcinoma” to describe malignant tumors, a term translated by the Roman physician Celsus (28–50 BC) into the Latin word “cancer”. Both terms mean crab, probably due to the ramified aspect of tumors [1]. The incidence of cancer cases has increased in the modern age, most likely due to the significant increase of life expectancy [2]. Nowadays, it counts as the second leading cause of death globally [3]. Cancer treatment currently takes a multidisciplinary approach, including chemotherapy, which often becomes the first choice in advanced, metastatic cancers. Despite tremendous therapeutic success, chemotherapy is also associated with drug resistance and severe side effects [4], thus raising the necessity to introduce new more efficient and less toxic drugs. Such approaches include targeting photothermal therapy by arginine–glycine–aspartic-acid-functionalized nanomaterials [5], cold plasma therapy [6], targeting by radioisotopes nanomaterials [7], gene therapy [8], magneto-photo-thermal therapy [9], treatment by stem cells [10], and fabrication of new radiosensitizers [11].

Natural compounds have always played a significant role in the design of novel therapeutics due to their enormous structural diversity providing high screening hit rates against various diseases [12]. Triterpenoids are a large group of widely found natural compounds that are particularly prevalent in plants. They display a broad spectrum of biological and pharmacological activities, such as antiviral, anti-inflammatory, and antitumor, combined with low toxicity [13]. Due to the presence of easily transformable functional groups, natural pentacyclic triterpenoids display a high potential for semisynthetic approaches [14] and are actively used as promising structural platforms for the development of new drugs [15].

Oleanolic acid (OA) and its structural isomer ursolic acid (UA) are widely spread secondary metabolites found in many medicinal plants. UA can be employed to treat various cancers, inflammatory diseases, diabetes, Parkinson’s disease, Alzheimer’s disease, and hepatitis B and C. OA possesses numerous pharmacological activities, such as antimicrobial (against a wide range of pathogens), anti-oxidant, anti-tumor, anti-fungal, anti-diabetic, anti-osteoporosis, anti-obesity, lipid-lowering, immune-regulatory, and hepatoprotective effects, and has been shown to be clinically effective as an anti-inflammatory and antitumorigenic agent [16,17,18,19,20,21,22]. OA has been used as hepatoprotective drug [23] as well as a treatment against cancer, chronic kidney disease, and psoriasis [24]. The potential of the above-mentioned triterpenic acids as biologically active compounds is well studied, in contrast to the oleanonic and ursonic acids holding an oxo-group at the C-3 position.

**Figure 1:**
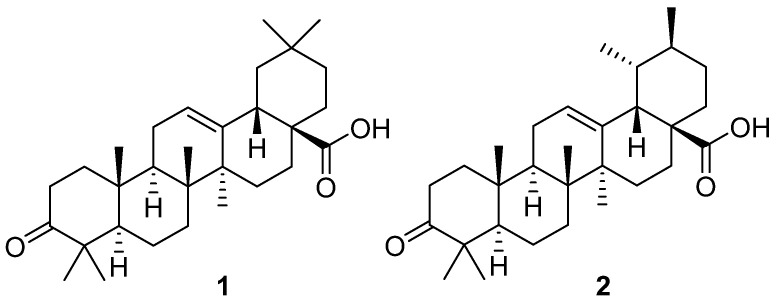
Structures of oleanonic **1** and ursonic **2** acids.

In recent years, several studies showed that the reported pharmacological effects of ursonic and oleanonic acids (Figure 1) were similar or stronger than their native counterparts [25,26]. Ursonic acid significantly decreased the proliferation of some types of cancer cells, such as A549 non-small lung cancer cells (IC_50_ 7.7 µM), HCT15 colon cancer cells (IC_50_ 4.6 µM), HONE-1 nasopharyngeal cancer cells (IC_50_ 5.2 µM), KB oral epidermoid cancer cells (IC_50_ 4.0 µM), and HT29 colorectal cancer cells (IC_50_ 6.3 µM); these data were comparable to the results recorded for ursolic acid [27,28]. It should be noted that ursonic acid [29] does not induce a cytotoxic effect against the NIH3T3 noncancer murine fibroblast cells (IC_50_ > 50 µM), while actively inhibiting several cancer cell lines such as MGC-803 gastric and PC3 prostate cancer cells (IC_50_ 13.6 µM to 26.5), thus exhibiting significant selectivity [29].

The presence of a piperazine moiety in a chemical compound has been associated with various pharmacological properties; many currently notable anticancer agents, including imatinib (STI571), dasatinib (BMS-354825), bosutinib (SKI-606), danusertib (PHA-739358), and VX-680 [30] contain a piperazine ring as part of their molecular structure. Studies have shown that the incorporation of a piperazine moiety could occasionally provide unexpected improvements in the bioactivity of the respective compounds [31,32,33]. The piperazine derivatives of triperpenic acids such as betulinic, oleanolic, ursolic, and glycyrrhetinic acid were found to exert numerous pharmacological effects. Bildziukevich et al. presented the synthesis of piperazine amide of betulinic acid, which showed medium to high cytotoxicity on T-lymphoblastic leukemia, breast adenocarcinoma, and cervical cancer cells (0.7–7.8 µM) [34]. Lan et al. described the synthesis and antiproliferative activity of 3,23-dihydroxy-17-piperazinyl betulinic amide against HeLa, MCF-7, HepG2, B16, and A375 cancer cell lines with IC_50_ values below 10 µM [35]. Sommerwerk et al. reported on the synthesis and antitumor activity of 2,3-di-O-acethyl-piperazinyl amides of oleanolic, ursolic, betulinic, and platanic acids, respectively, with significant cytotoxity against the human cancer cell lines: 518A2 (melanoma), HT29 (colorectal adenocarcinoma), MCF7 (breast adenocarcinoma), A549 (lung adenocarcinoma), A2780 (ovarian carcinoma), and 8505C (thyroid carcinoma), with EC_50_ values ranging from 1.4 to 10.5 µM [36]. Giniyatullina et al. described the synthesis and antitumor activity of betulinic acid *N*-methyl-piperazinyl amide against HOP-62 (NCS lung cancer); HCC-2998, HCT-116, KM-16 (colon cancer); MDA-MB-486 (breast cancer); OVCAR-3 (ovarian cancer); M14 (melanoma); and 768-0 (renal cancer) cancer cells [37].

Molecular hybridization is one of the most effective approaches to finding new drug platforms by combining several bioactive units in a single structure. The conjugates that resulted from the coupling of acetylated piperazinyl amide spacered oleanolic and ursolic acids, respectively, with meta or para substituted carboxylated malachite green analogs were highly cytotoxic on several human tumor cell lines, in particular MCF-7 human breast carcinoma, with EC_50_ 0.7 μM [38]. Betulinic acid piperazinyl amide was found to exert strong cytotoxicity against the CCRF-CEM cell line, with an IC_50_ value of 0.8 µM but rather low selectivity [39]. Among the studied *N*-methylpyperazinyl amides of betulinic, platanic, glycyrrhetic, oleanolic, ursolic, and moronic acids, respectively, the highest activity against *S. aureus* was observed for a betulonic acid derivative [40]. The incorporation of a piperazine moiety while retaining the polar C3 group could significantly improve the anticancer activity against breast and gastric cancer cell lines [41,42,43].

Several authors reported that chalcones (1,3-diaryl-2-propene-1-ones), obtained by Claisen-Schmidt reaction, have a variety of activities. When introduced on oleanane and ursane templates, respectively, they lead to derivatives of oleanolic acid and ursolic acid, respectively, substituted with benzylidene fragments at the C2 position in the A-ring, which also contains a 1-en-3-one structure; the resulting derivatives display increased anticancer [41,44,45,46] and antidiabetic activities [47].

In the present work, the synthesis of novel hybrid derivatives bearing chalcone and *N*-ethyl-pyperazinyl amide functions in the frames of oleanonic and ursonic acids, respectively, is described. The novel compounds were investigated in terms of their anticancer cytotoxic activity and molecular mechanisms.

## 2. Results and Discussion

### 2.1. Chemistry

**Scheme 1:**
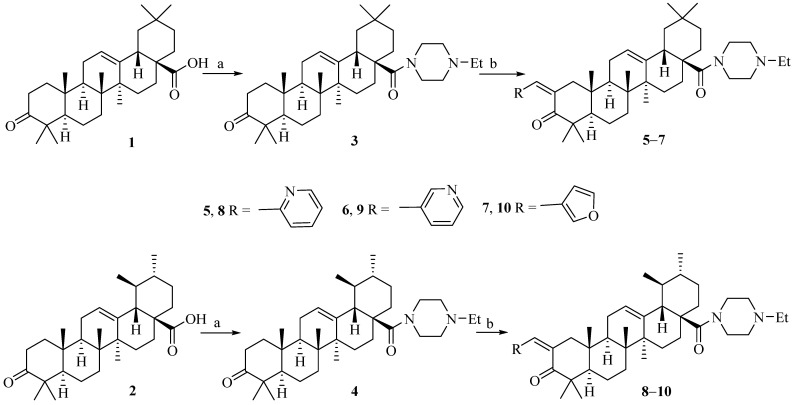
Synthesis of oleanonic and ursonic acids’ derivatives **3**–**10**: (a) *i* (COCl)_2_, CH_2_Cl_2_, rt, 3 h; *ii N*-ethylpiperazine, CH_2_Cl_2_, Et_3_N, ∆, 5 h; (b) 2- or 3-pyridinecarboxaldehyde or furfural, 40% KOH, EtOH, rt, 24 h.

Oleanonic **1** and ursonic **2** acids were used as parent compounds in the synthesis of new hybrid derivatives by introducing of *N*-ethylpiperazinyl-amide at C28 and a “chalcone-like” fragment at C2 position. Acylation of *N*-ethyl-piperazine by oleanonic or ursonic acids’ chlorides led to amides **3** and **4** in yields of 94% and 91% (Scheme 1). The Claisen-Schmidt condensation of *N*-ethyl-piperazinyl amides **3** and **4** with 2- or 3-pyridinecarboxaldehydes or furfural in EtOH in the presence of 40% KOH at room temperature afforded C2-nicotinoylidene and furfurylidene **5**–**10** derivatives in good yields (91–97%) (Scheme 1). The structure of compounds was ascertained by use of spectral methods. The formation of amide groups at C28 of compounds **3** and **4** were confirmed by characteristic signals of carbons at δ_C_ 174.87 and 175.15 ppm in the ^13^C NMR, correspondently. The new signals of -CH_2_- groups of *N*-ethylpiperazine ring resonated at δ_C_ 52.23–55.40 ppm.

In the ^13^C NMR spectra of the chalcone derivatives **5**–**10,** the shifts of signals of C3 carbonyl atoms were observed at δc 207.00–208.70 ppm, and the signals of the aromatic fragments resonated at δ_C_ 112.18–155.61 ppm and δ_H_ 7.05–8.64 ppm (^1^H NMR) (see Supplementary Material, Figures S1–S8).

### 2.2. Biological Activity

#### 2.2.1. NCI-60 Anticancer Drug Screening

Compounds **3**–**10** were selected by the National Cancer Institute (NCI, Bethesda, MA, USA) Developmental Therapeutic Program (DTP) and tested at one dose assay (10^−5^ M) toward a panel of approximately sixty cancer cell lines representing different cancer types; the results for each compound are reported as the percent growth (GP %) of treated cells compared to untreated control cells (negative numbers indicate cellular death). The range of percent growth shows the lowest and the highest percent growth found among the different cancer cell lines (Table 1 and Supplementary Material, Figures S9–S16). The experimental data allow the assessment of the cytostatic effect, indicated by growth inhibition values ranging between 0 and 100, as well as of the cytotoxic effect, indicated by negative values. The NCI-60 anticancer drug screening was developed in the 1980s as an in vitro tool able to provide reliable information in the field of cytotoxic drugs [48], particularly those of natural origin [49].

Based on the results of one dose primary screening, *N*-ethylpiperazinyl amide of oleanonic **3** acid showed moderate growth inhibition activity. Compound **3** was selectively active against leukemia cancer cell lines with growth percentages ranging from −35.82 to 37.47%, while the highest activity was recorded against HL-60(TB) cell lines (−35.82%). A pronounced selectivity against A498 renal cancer cell line (−7.95%) was also noticed, while the introduction of the *N*-ethylpiperazinyl amide function into the ursonic acid core led to an increased antiproliferative effect. Compound **4** showed a broad spectrum of cell inhibition against all 60 human cancer cell lines with a reduction in growth ranging from −85.67 to −41.54%, and displayed the highest cytotoxicity against MALME-3M, LOXIMVI, SK-MEL-5, and SK-MEL-28 melanoma cell lines, as well as SF-539 CNS cancer cell lines, exhibiting a reduction in cell proliferation of −85.67, −85.17, −84.06, −64.66%, and −97.21%, respectively.

Compounds **5** and **8** holding the 2-pyridinoylidene unit at C-2 position of the triterpenoid core do not exhibit antiproliferative effects.

Anticancer data revealed a reduction of cell growth ranging between −90.15 and 83.75%, and between −38.20 and 65.71%, respectively, for 3-pyridinoylidene derivatives of oleanonic **6** and ursonic **9** acids. The cell lines most sensitive to the antiproliferative effects of compound **6** were U251 non-small cell lung cancer (90.15% cell death) and SK-MEL-5 melanoma (88.15% cell death), while for compound **9** the highest percentage of cell death was recorded against the HL-60(TB) leukemia cells (38.20% cell death).

The 2-furfurylidene derivatives **7** and **10** exerted cytostatic effects only against leukemia cell lines (HL-60(TB) and SR, respectively) with growth percentages of 16.53 and 31.17%, respectively.

The experimental data showed that the cytotoxic effect of the studied chalcone-piperazinyl amides were directly dependent on the arylidene type moieties. Thus, compounds **5** and **8** with 2-pyridinoylidene substituents, and **7** and **10** with furfurylidene groups, do not induce any significant antiproliferative activity. The introduction of a 3-pyridinoylidene group led to the compounds with antiproliferative activity; 3-pyridinoylidene oleanane **6** and ursane **9** were equally cytotoxic against **4** and **5** cell lines, respectively.

Collectively, three of the new compounds (**4**, **6,** and **9**) possessed considerable activity against all tested human tumor cell lines and were selected for the advanced assay against a panel of approximately sixty tumor cell lines at 10-fold dilutions of five concentrations (100 µM, 10 µM, 1 µM, 0.1 µM, and 0.01 µM) [50,51,52,53,54]. The mean GI_50_ (molar concentration of the compound that inhibits 50% net cell growth), TGI (molar concentration of the compound leading to the total inhibition), and LC_50_ (molar concentration of the compound leading to 50% net cell death (presented in negative logarithm)) values across each cell line are shown in Table 2. These compounds exhibited significant antiproliferative effects towards the tested human cancer cell lines; the highest cytotoxic activity in the five-dose testing was reported for compound **4**, which revealed submicromolar GI_50_ values (0.70–0.99 μM) against the most sensitive cell lines. The cytotoxic activities, expressed as LC_50_ values, for compounds **4**, **6**, and **9** against the most sensitive cancer cell lines were also high, ranging between 1 and 6 μM (Table 2 and Supplementary Material Figures S17–S19). Compound **4** showed a broad spectrum of growth inhibition activity (GI_50_ < 2 μM) against all human tumor cells with average GI_50_/TGI/LC_50_ values of 1.03/2.74/12.31 µM. Compounds **6** and **9** also revealed a broad spectrum of growth inhibition activity (GI_50_ < 4 μM) against all human tumor cells with average GI_50_/TGI/LC_50_ values of 1.52/6.29/32.36 µM for compound **6** and 1.93/5.29/37.95 for compound **9** (Table 2). Mean GI_50_ values for these compounds in comparison with standard anticancer agents, doxorubicin and 5-fluorouracil [55], are displayed in Table 2.

The selectivity index (SI), calculated as the ratio between the full panel MG_MID_60_ (μM) of compounds **4**, **6**, and **9** by their respective subpanels MG_MID of the cell line (μM), was introduced as a quantification tool for the selectivity of each compound in terms of anticancer effect (Table 3). Ratios between 3 and 6 are indicators of moderate selectivity, and ratios greater than 6 indicate high selectivity, while ratios below 3 indicate nonselective compounds [56]. In this context, compound **4** was found to be nonselective for all GI_50_, TGI, and LC_50_ levels (selectivity indexes 0.81–1.11, 0.62–1.41, and 0.45–2.29, respectively); derivatives **6** and **9** demonstrated a moderate selectivity profile toward some individual cell lines at the LC_50_ level. Thus, selectivity indexes (SI) of compound **6** at the LC_50_ level were 3.52 for melanoma, and 4.04 for prostate cancer; for compound **9,** an SI value of 4.11 for prostate cancer was revealed.

#### 2.2.2. Compounds **4** and **6** Induce Apoptosis-Related Nucleus Changes

**Figure 2:**
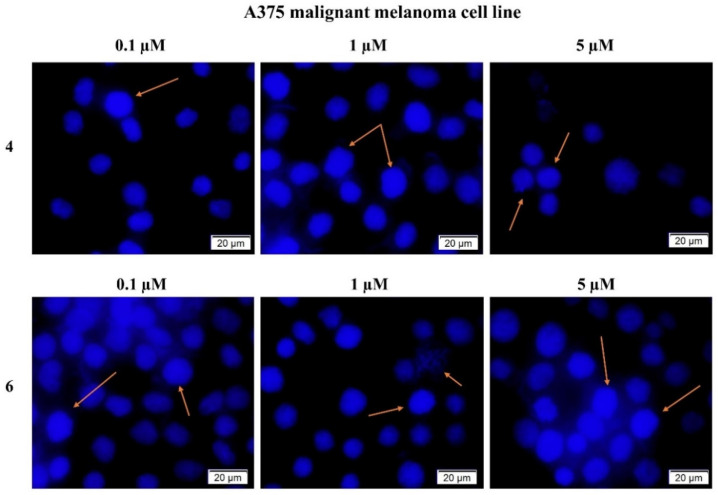
Effect of compounds **4** and **6** (0.1, 1, and 5 μM) after 24 h of stimulation on the nuclear morphology of A375 cells—DAPI staining. The white arrows indicate apoptotic features. The scale bars represent 20 µm.

Compounds **4** and **6** were further subjected to DAPI (4′,6-diamidino-2-phenylindole) staining in order to provide a more detailed picture of their antiproliferative and cytotoxic mechanisms. Prior to this assay, compound stability in cell culture media was evaluated after 72 h, using a thin-layer chromatography (TLC)-based protocol, with the necessary modifications [57,58]. Developed plates (using three different revealing agents) showed no other spots except the one corresponding to the original compound, suggesting that compounds **4** and **6** show no signs of decomposition in culture media for 72 h. Considering that compound **4** (highest overall cytotoxic activity) exhibited the highest overall cytotoxic activity on the melanoma cell line panel, the DAPI test was conducted on three human melanoma cell lines: A375, RPMI, and SK-MEL-28. According to literature data, the doubling time for A375 is about 6–12 h; for SK-Mel 28, 27 h; and for RPMI, about 60 h [59,60]. Considering this aspect, in addition to SK-Mel 28, the A375 and RPMI cell lines were chosen for this purpose to observe the effect of the tested compounds on tumor cells with different multiplication rates. Compounds were applied in three concentrations: 0.1, 1, and 5 μM, respectively. The mid 1 μM concentration was selected as the nearest integer value to the average GI_50_ of compounds **4** (highest activity) and **6** for the melanoma cell panel (0.93 μM, 1.54 μM). The other two concentrations, 0.1 and 5 μM, were tested to see if a concentration-dependent effect is observable around the mid GI_50_ value. All cells were treated with the nuclear-specific DAPI stain which binds selectively to DNA and emits a characteristic blue fluorescence under UV radiation [61]. DAPI staining is a simple and inexpensive yet highly specific and quantifiable procedure for nuclei visualization that has become frequently employed in apoptosis detection [62]. The compounds induced the dose-dependent nuclei condensation of the A375 melanoma cells; thus, specific apoptotic features such as chromatin condensation, membrane blebbing, and nuclei fragmentation indicated by orange arrows in Figure 2 can be noticed following the treatment with both compounds, even at the lowest tested concentration (0.1 μM). However, the most significant changes were recorded when the 5 μM concentration was applied. By contrast, DMSO, which was used as solvent, induced insignificant nucleus condensation only when the highest concentration was tested.

**Figure 3:**
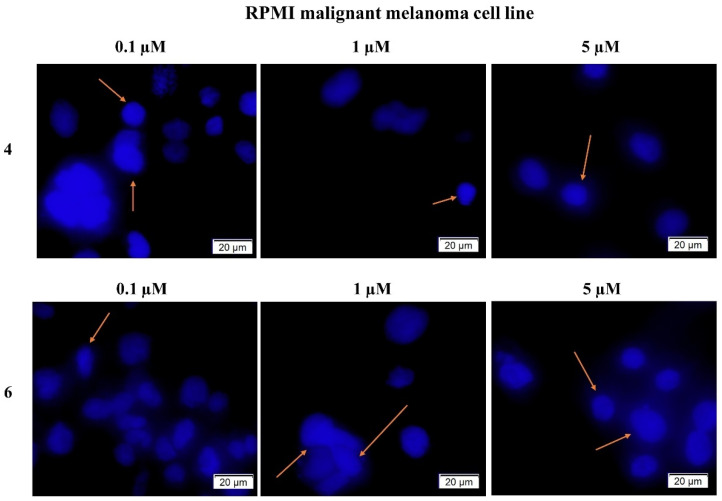
Effect of compounds **4** and **6** (0.1, 1, and 5 uM) after 24 h of stimulation on the nuclear morphology of RPMI cells—DAPI staining. The white arrows indicate apoptotic features. The scale bars represent 20 µm.

Similar observations were recorded on RPMI melanoma cells (Figure 3), indicating apoptotic cell death. Compound **4** induced massive chromatin condensation and visible nuclear fragmentation at 0.1 µM and 1 µM, respectively, while the highest concentration led to the blebbing of the nuclear membrane. Compound **6** induced differential effects on the nuclear morphology depending on the tested concentration as follows: (i) nuclear fragmentation at 0.1 µM; (ii) chromatin condensation at 1 μM; and (iii) apoptotic bodies at 5 μM, all changes being indicated by orange arrows in Figure 3.

**Figure 4:**
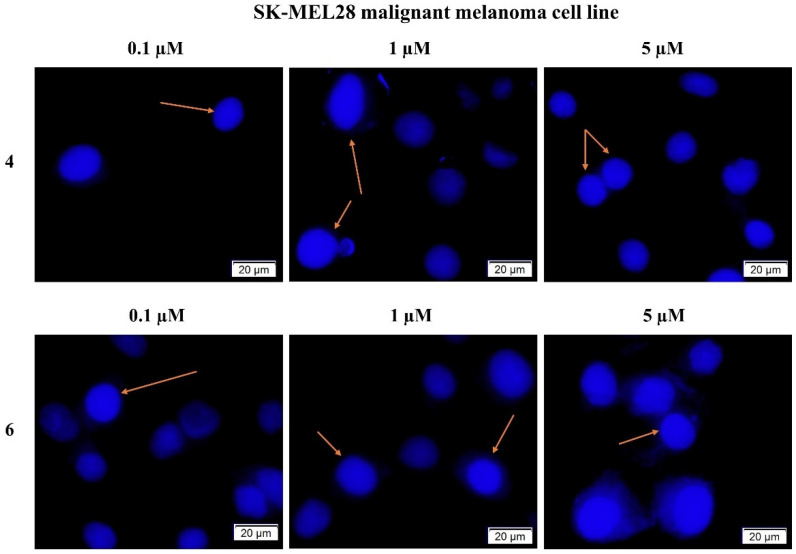
Effect of compounds **4** and **6** (0.1, 1, and 5 uM) after 24 h of stimulation on the nuclear morphology of SK-Mel28 cells—DAPI staining. The white arrows indicate apoptotic features. The scale bars represent 20 µm.

In SK-MEL28 malignant melanoma cells (Figure 4), the 0.1 μM compound **4** induced an intense condensation of the cell nuclei; when applied as a 1 μM sample, the chromatin condensation is accompanied by the formation of apoptotic bodies (white arrows), while at 5 μM, incipient nuclear fragmentation can be noticed. Similar morphologic events have been induced by compound **6,** where a dose-dependent effect was recorded in terms of membrane blebbing, nuclear condensation, and fragmentation (highlighted by orange arrows in Figure 4).

#### 2.2.3. Compounds **4** and **6** Induce Changes in Cell Morphology and Confluence

**Figure 5:**
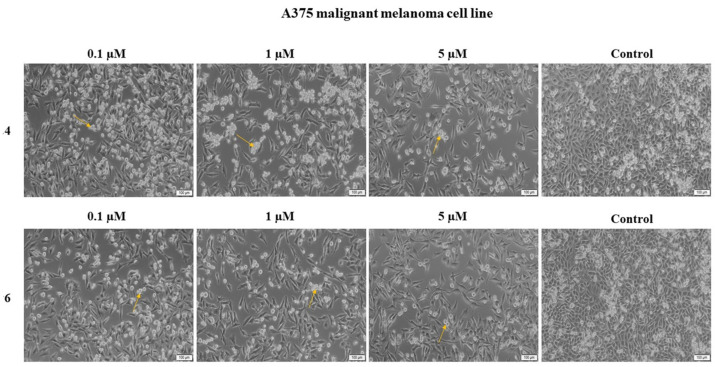
Morphological aspect of the A375 cells following the 24 h treatment with compounds **4** and **6** (0.1, 1, and 5). The arrows indicate the round and detached cells suggesting cell death. The scale bars represent 100 μm.

A morphological examination of the tested cells was conducted in order to provide additional data for the investigated compounds in terms of their biological activity. In A375 cells (Figure 5), the 0.1 μM compound **4** caused cell death as indicated by the agglomeration of round and floating cells (yellow arrows). When used in higher concentrations (1 and 5 μM), the cell detachment is accompanied by a visible loss of cell confluence (viable cells) as compared to control (untreated) cells. Compound **6** induced a noticeable decrease in cell confluence, as well as cell roundness and detachment from the plate, but the respective effects were similar for all concentrations; all these changes are signs of apoptosis-induced cytotoxicity [63].

**Figure 6:**
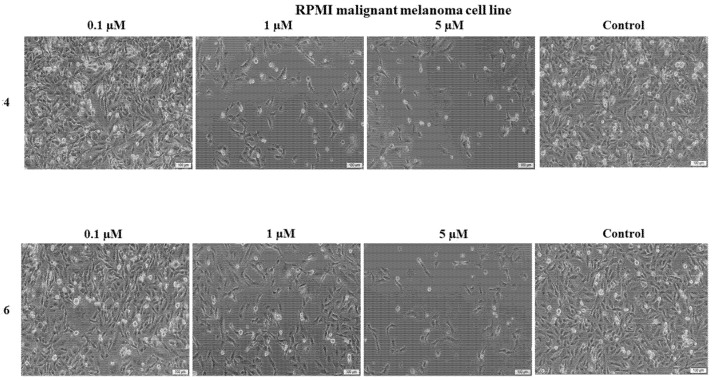
Morphological aspect of the RPMI cells following the 24 h treatment with compounds **4** and **6** (0.1, 1, and 5 μM). The arrows indicate the round and detached cells suggesting cell death. The scale bars represent 100 μm.

**Figure 7:**
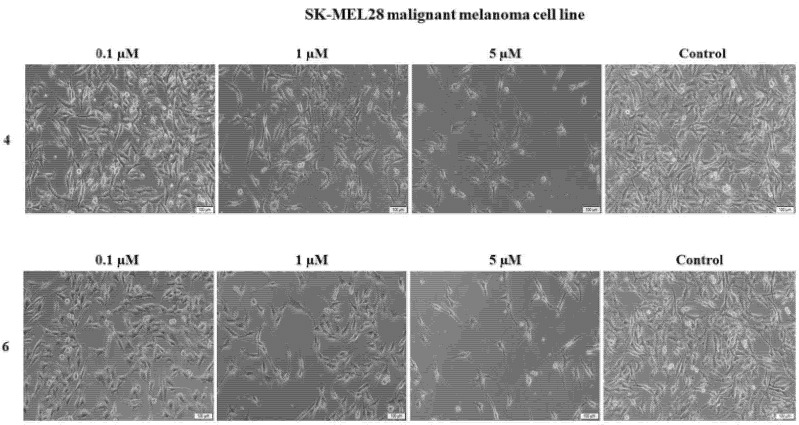
Morphological aspect of the SK-MEL28 cells following the 24 h treatment with compounds **4** and **6** (0.1, 1, and 5 μM). The arrows indicate the round and detached cells suggesting cell death. The scale bars represent 100 μm.

In RPMI melanoma cells (Figure 6), both compounds induced similar effects; the 0.1 μM sample did not induce any changes in cell confluence or shape compared to control, while for the 1 and 5 μM samples, respectively, a significant and concentration-dependent loss of cell confluence (viable cells) and number can be noticed. In SK-MEL28 cells (Figure 7), the compounds were active starting from the lowest concentration (0.1 μM) by decreasing melanoma cells confluence; the effect was significantly higher at 1 and 5 μM. In all three cases mentioned above, there is an apparent large number of round detached dead cells in the control group. This occurrence is due to the accentuated growth of the cells and the decrease of the space and the necessary nutrients in the well. This phenomenon is more pronounced in the case of the A375 cell line, with the shortest doubling time (6–12 h) having an aggressive profile and a marked rapid growth (Figure 5).

Apoptosis is an orchestrated and complex cell death process which takes place in both physiological and pathological states; in cancer, the normal apoptotic process is diminished, altering the balance between living and dead cells and resulting in anomalous cell proliferation [64]. Apoptosis targeting is currently considered an effective approach to fight all types of cancer as well as drug resistance and a useful tool in drug discovery and validation [65]. However, the apoptotic process should be clearly differentiated from necrosis; while apoptosis is an active, energy-consuming, and programmed process that avoids triggering inflammation, necrosis is a passive and accidental cell death usually resulting from external stimuli such as trauma and is accompanied by uncontrolled inflammatory reactions [66]. The DAPI staining highlighted nuclear changes associated with apoptosis, such as shrunken and marginated nuclei, in clear contrast with the normal, large nuclei found in untreated cells. Our recorded data are consistent with previous studies that reported the antiproliferative effect of 3-oxo oleanolic acid derivatives on melanoma [26]. Later, Li et al. synthesized a series of 15 conjugates of oleanonic acid, which showed excellent cytotoxicity against the A375-S2 melanoma cell line [67]. An excellent review published in 2020 by Son and Lee highlighted the remarkable cytotoxic activity of ursonic acid against numerous cancer cell lines; in addition, the study reveals that ursonic acid induces apoptosis through the mitochondrial intrinsic mechanism, which was also identified for some of its semisynthetic derivatives [25]. Interestingly, for some types of cancer, the cytotoxic activity of ursonic acid occurred at significantly lower concentration than for the more studied ursolic acid, and even certain standard anticancer drugs such as cisplatin.

#### 2.2.4. rtPCR Assay Highlights Biological Activity at Gene Level

**Figure 8:**
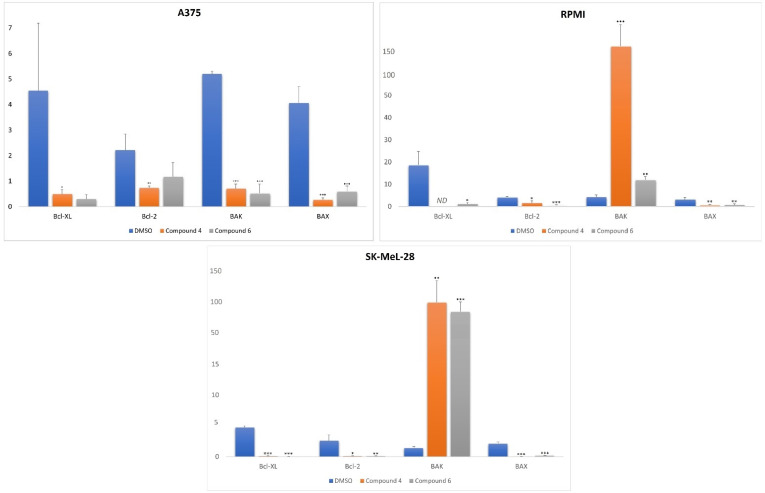
Expression of the BAK, Bax, BCL-2, and BCL-XL genes treated with 1 μM solutions of compounds **4** and **6** and DMSO. Data are shown on as mean 2^∆∆Ct^ values with one standard deviation; (*) indicates significant differences as compared to the reference group (Student’s *t* test, *** *p* < 0.001, * *p* < 0.01, ** *p* < 0.05). *ND*—not determined.

Apoptosis at the cellular level can be initiated through the mitochondrial intrinsic pathway, which involves the Bcl-2 family of genes that includes the anti-apoptotic Bcl-XL and Bcl-2, as well as the pro-apoptotic Bak and Bax members; the balance between the expressions of anti- and pro-apoptotic proteins ensures physiological cell death [68]. In cancers, the alterations occurring in gene expressions due to down- or up-regulations may result in delayed apoptosis and malignant proliferation. Thus, active compounds that modify the abnormal expression of genes involved in the apoptotic process may successfully act as anticancer agents [69]. We tested the effect of compounds **4** and **6** on the expression of anti-apoptotic (Bcl-XL and Bcl-2) and pro-apoptotic (Bak and Bax) genes (Table 4, Figure 8). In all three cell lines, both compounds induced the down-regulation of anti-apoptotic Bcl-XL and Bcl-2, as well as of pro-apoptotic Bax genes; however, in RPMI and SK-Mel-28 cells, both compounds induced the very strong up-regulation of the executioner pro-apoptotic Bak gene that permeabilizes the mitochondrial outer membrane, releasing activators of apoptosis effector caspases [70].

The up-regulation of pro-apoptotic Bak gene combined with the down-regulation of the pro-survival Bcl-XL and Bcl-2 genes presumably caused altered ratios between the pro-apoptotic and anti-apoptotic proteins’ levels, leading to overall stimulated apoptosis. The intervention of pentacyclic triterpenes on genes involved in the intrinsic mitochondrial apoptotic pathway has been previously reported; the diamine-(PEG)ylated oleanolic acid clearly up-regulated caspase-8, caspase-9, caspase-3, and Bak, and down-regulated Bcl-2 [71]. Conversely, 3-O-α-L-arabinosyl oleanolic acid caused up-regulation of Bax [72]. Similarly, ursolic acid reduced the expression of Bcl-2 and increased the level of the apoptotic protein Bax [73]. Collectively, we may conclude that our two compounds, in line with previously reported triterpene derivatives, induce an apoptotic cancer cell death through altering the ratio between the proapoptotic and antiapoptotic proteins. Of course, given the case of A375 cells, in which both pro and anti-apoptotic genes were downregulated but in previous determinations signs of apoptosis were observed, we can deduce that the pro-apoptotic mechanism of the tested compounds can be more complex, acting beyond the modulatory effect of pro- and anti-apoptotic genes.

### 2.3. Compounds ***4*** and ***6*** Exhibit In Silico Bcl-XL Inhibition

Currently, computational techniques are an essential helping hand used to shorten the time needed to understand the action mechanisms for compounds with quantifiable pharmacological effect [74,75,76]. Molecular docking is a versatile technique that uses the 3D structure of the biological target (determined experimentally by X-ray crystallography, NMR, or computer) to dock the candidate molecules and classify them based on their binding affinity, calculated using a scoring function, or complementarity, in the conformational space of the target protein active site [77].

Taking into consideration the recorded rtPCR results, a molecular-docking-based protocol was applied in order to determine the targeted in silico inhibitory potential of compounds **4** and **6** against protein targets involved in apoptotic cell death signaling pathways, such as: apoptosis regulators Bcl-X (Bcl-XL) and Bcl-2 (Bcl-2), induced myeloid leukemia cell differentiation protein (Mcl-1), phosphatidylinositol 4,5-bisphosphate 3-kinase catalytic subunit gamma isoform (PI3Kγ), dual specificity mitogen-activated protein kinase kinase 1 (MEK1), mammalian target of rapamycin (mTOR). Docking results for compounds **4** and **6** are displayed in Table 5.

The obtained results reveal that compounds **4** and **6** registered good docking scores compared to those of the co-crystalized ligand (−8.5 kcal/mol, −9.2 kcal/mol), for the Bcl-XL protein (PDB ID: 2YXJ). Comparing the two compounds, it appears that compound **6** was slightly more active. This detail can be explained by analyzing the orientation of the two compounds in the binding pocket of the target protein.

**Figure 9:**
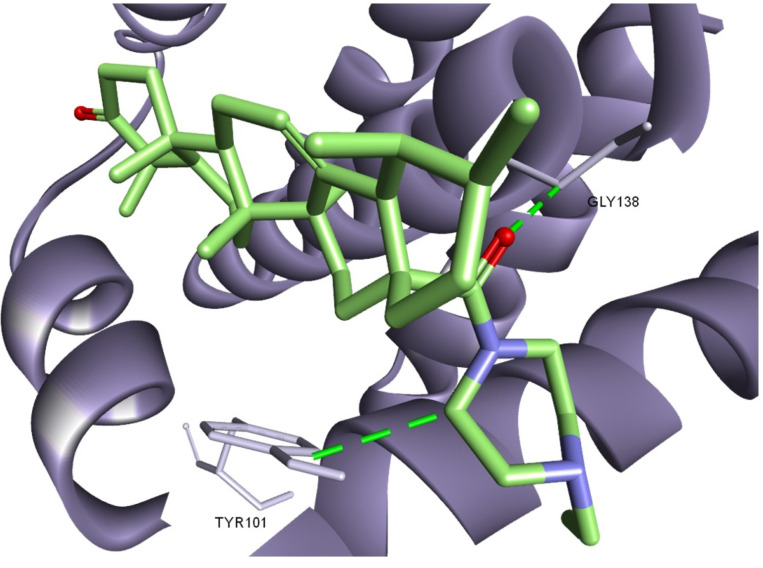
Structure of Bcl-XL (2YXJ) in complex with compound **4** (green); HB interactions are depicted as green dotted lines; interacting amino acids are shown as light purple sticks.

**Figure 10:**
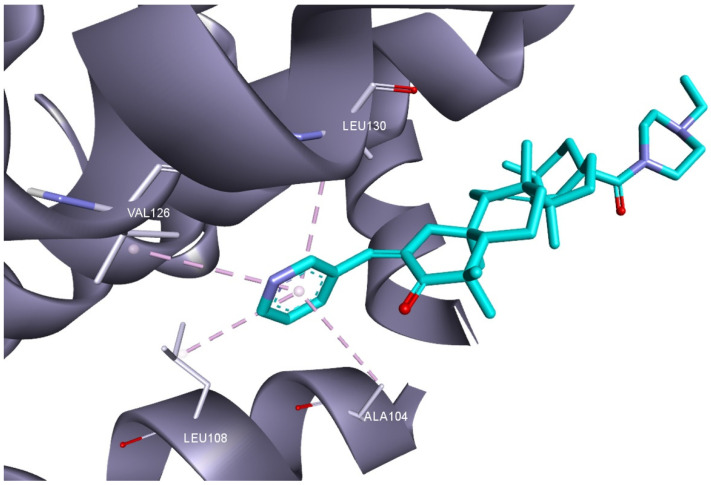
Structure of Bcl-XL (2YXJ) in complex with compound **6** (blue); hydrophobic interactions are depicted as purple dotted lines; interacting amino acids are shown as light purple sticks.

The Bcl-XL binding site contains four hydrophobic pockets, with which only BH3 proteins interact. The co-crystallized inhibitor ABT-737 is structurally designed to interact with the hydrophobic pockets p2 and p4 [78]. With this detail in perspective, compound **4** does not form hydrophobic interactions, but interacts within the binding site with Tyr101 and Gly138 (Figure 9), by forming two unconventional HBs (O–H–C). At the same time, compound **6** occupies one of the four hydrophobic pockets, similar to the inhibitor ABT-737, interacting with nearby amino acids such as Ala104, Leu108, Val 126, and Leu130 (Figure 10). This conformational orientation seems to be the reason why compound **6** achieved a better score than compound **4**. Our findings are in line with a previously published study that reported the in silico inhibitory potential of triterpenes (ursolic acid and oleanolic acid) to target Bcl-XL. The study also revealed that results obtained by the two compounds scored better than the Bcl-XL inhibitor ABT-737 [79]. These findings suggest that compounds **4** and **6** could also exert a pro-apoptotic effect by targeting the anti-apoptotic protein Bcl-XL.

### 2.4. Effect of Compounds ***4*** and ***6*** on Normal and Tumor Angiogenesis Process by CAM Assay and Irritation Potential Determination Using the HET-CAM Assay

Angiogenesis or tumor vascularization is a complex process, consisting in the emergence of new blood vessels, that involves several signaling pathways and may evolve differently in different cancer subtypes or even within the same tumor [80]. Anti-angiogenic therapies have been investigated and several drugs were introduced as cancer treatments, but so far, the clinical outcomes were modest and resulted in poor survival benefits [81].

**Figure 11:**
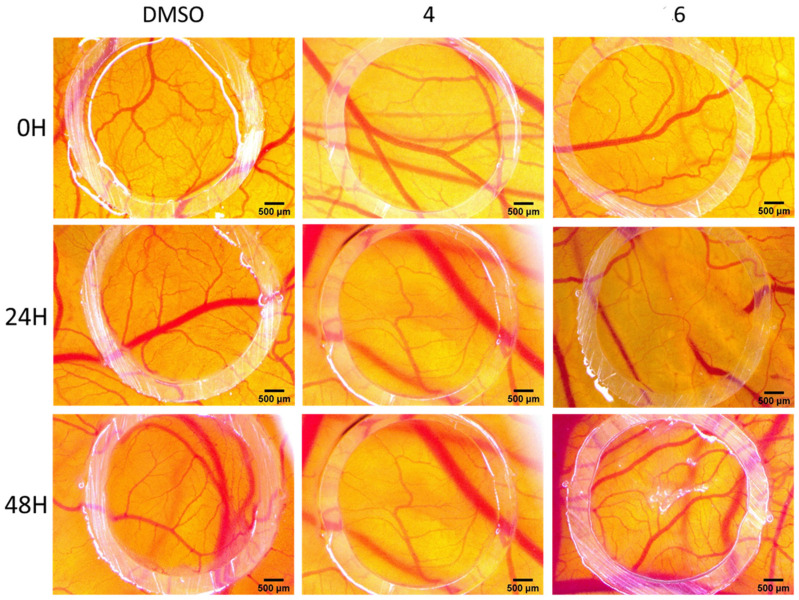
The angiogenesis assessment of compounds **4** and **6**, respectively, using the CAM assay. Stereomicroscope images represent show daily modification upon the treated vascular plexus; scale bars represent 500 µm.

The investigation of potential antiangiogenic mechanisms was achieved by using the choryoallantoic membrane (CAM) assay, an excellent Ex vivo test which does not imply ethics assessments and is relatively simple, rapid, low cost, and less time consuming. CAM test also allows the assessment of a drug’s activity on tumor proliferation and distant metastasis, as well as drug toxicity [82]. The compounds were applied as 1 µM solutions in 0.5% aqueous DMSO on CAM and monitored from EDD7 to EDD10 in order to identify any potential inhibition of the angiogenesis process. After the application of compound **4** on CAM, no alterations were noticed in terms of angiogenesis during 72 h post-treatment, thus indicating the lack of anti-angiogenic properties (Figure 11). No anomalies were noticed in the angiogenic process after treatment with 0.5% DMSO in distilled water used as control.

Compound **6** did not significantly alter the angiogenesis process within 48 h post treatment; however, after 24 h, a few areas with modified vascular branching pattern were identified, with a reduced number of new forming vessels, thus indicating a weak antiangiogenic process.

To the best of our knowledge, no other 3-oxo-derivatives of ursolic acid were previously investigated in terms of angiogenic effects. If we compare compound **4** to ursolic acid, two hypotheses are plausible: either the compound does not intervene in the angiogenic process, or its effect depends on its concentration and the CAM assay test should be repeated at various concentrations. Similarly, compound **6** only slightly induced an antiangiogenic effect, unlike the native OA and some of its derivatives such as 2-cyano-3, 12-dioxoolean-1, and 9-dien-28-oic acid (CDDO), which, due to their strong anti-angiogenic activity in murine cancer models, are currently under investigation in phase I clinical trials [83]. Interestingly, Sun et al. synthesized bioconjugates of oleanonic acid with the semibenzoquinone jacaranone, which showed significant anti-angiogenic properties and selective cytotoxic effects against melanoma cells; the authors predicted certain anti-angiogenesis-related targets [84]. This was the only study found in the literature that mentions the anti-angiogenic effect of oleanonic acid; however, a potential explanation for this behavior is the ability of oleanonic acid and its derivatives to inhibit NO production, which promotes angiogenesis [85]. If one tries to draw a parallel with the 3-oxo-derivative of another important triterpenic betulinic acid, one can notice that, despite substantial evidence of strong antiproliferative effects of both betulonic acid and its semisynthetic derivatives, as highlighted by Lombrea et al., no studies have reported the occurrence of anti-angiogenic activity [86]. Therefore, we might conclude that the anticancer effect of our two compounds does not involve their interference with the normal/pathological angiogenic process.

**Figure 12:**
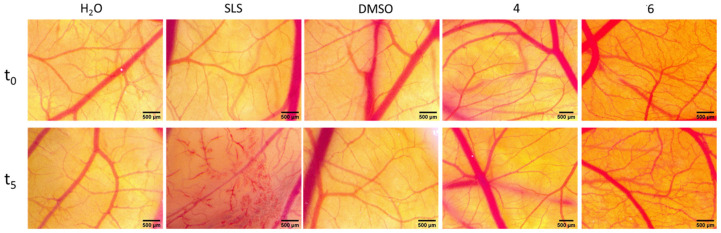
The irritation potential assessment using the HET-CAM method. Stereomicroscope images show the in face chorioallantoic membrane before (**t_0_**) and 300 sec after application (**t_5_**) of 300 µL of compounds **4** or **6** in concentration of 1 µM and control samples (distilled water H_2_O as negative control, SLS 0.5% as positive control, and DMSO 0.5% as solvent control); scale bars represent 500 µm.

The toxicity of the compounds was investigated by means of HET-CAM assay (Figure 12), which is a reliable Ex vivo test used to mimic in vivo inflammatory reactions and recognized as a predictive model for the irritant effect on conjunctival tissues [87]. In this assay, the irritation potential may range from 0 to 21, with 0 being non-irritant and 21 being the most irritant. The strong irritant sodium laurylsulphate (SLS) 0.5% was used as reference, while distilled water represented the non-irritant sample. The irritation score was established according to Luepke scale: 0–0.9 indicates non-irritant, 1–4.9 indicates weakly irritant, 5–8.9 indicates moderately irritant, while 9–21 means strongly irritant (Table 6) [88,89]. Several parameters were assessed in order to quantify the irritative potential of tested compounds: haemorrhage, lysis, and coagulability; no such phenomena occurred during our investigation. Therefore, a score of 0 was attributed to the investigated compounds, as well as to the negative control, thus classifying them as non-irritant. By contrast, the irritation score attributed to the positive control sodium laurylsulphate was 17.03, thus qualifying it as a strong irritant; the solvent alone induced a slight irritation effect, with a score of 0.68. The HET-CAM assay revealed the biocompatibility of the tested compounds with mucosal tissues, thus making them safe for the purpose of therapeutic applications.

## 3. Materials and Methods

### 3.1. Chemistry

#### 3.1.1. General

^1^H and ^13^C NMR spectra were recorded on a Bruker AM-500 and Bruker Avance-III, at 500 and 125.5 MHz, respectively (Bruker, Billerica, MA, USA), in CDCl_3_, internal standard—tetramethylsilane, at the Center for the Collective Use “Chemistry” of the UIC UFRC RAS and RCCU “Agidel” of the UFRC RAS. Mass spectra were obtained on a liquid chromatograph–mass spectrometer LCMS-2010 EV (Shimadzu, Kyoto, Japan). Melting points were detected on a Rapido PHMK05 microtable (Nagema, Dresden, Germany). Optical rotations were measured on a Perkin-Elmer 241 MC polarimeter (PerkinElmer, Waltham MA, USA) in a tube length of 1 dm. Elemental analysis was performed on a Euro EA-3000 CHNS analyzer (Eurovector, Milan, Italy), and the main standard was acetanilide. Thin-layer chromatography analyses were performed on Sorbfil plates (Sorbpolimer, Krasnodar, Russian Federation), using the solvent system chloroform–ethyl acetate, 40:1. Substances were detected by a 10% solution of sulfuric acid solution with subsequent heating at 100–120 °C for 2–3 min. All chemicals were of reagent grade (Sigma-Aldrich, St. Louis, MO, USA). Oleanonic **1** and ursonic **2** acids were obtained according to [90,91].

#### 3.1.2. Synthesis of Compounds **3**, **4**

To a solution of compound **1** or **2** (1 mmol, 0.46 g) in CH_2_Cl_2_ (20 mL), (COCl)_2_ (3 mmol; 0.26 mL) was added and stirred at room temperature for 2 h. The mixture was concentrated to dryness under reduced pressure, and the resulting acid chloride was dissolved in CH_2_Cl_2_ (10 mL), and 3 drops of Et_3_N and 1.5 mmol of the *N*-methylpiperazine were added. After completion of the reaction (TLC control) the organic layers were treated with 5% HCl (3 × 50 mL) until neutral pH, dried over CaCl_2_, and evaporated under reduced pressure. The solvent was removed in vacuo and the product was purified by column chromatography on Al_2_O_3_ eluting with n-hexane-EtOAc (from 40:1 to 1:1).

*N*-(3-oxo-olean-12-en-28-oyl)-ethylpiperazine **3**. Yield: 0.43 g (94%); m.p. 150 °C; [α]*_D_^20^* + 107 (c 0.05, CHCl_3_); 1H NMR (CDCl_3_, δ, ppm): 0.77, 0.87, 0.91, 1.01, 1.01, 1.06, 1.12, 1.12 (24H, 8s, 8CH_3_), 1.13–2.11 (23H, m, CH, CH_2_), 2.22–2.40 (2H, m, CH_2_), 3.01 (4H, br.s, 2CH_2_), 3.58–3.71 (4H, m, 2CH_2_), 5.26 (1H, br.s, H-12); ^13^C NMR (CDCl_3_, δ, ppm): 11.88, 15.00, 16.26, 17.45, 19.61, 21.29, 21.48, 23.43, 23.46, 24.08, 25.89, 26.43, 26.54, 27.92, 29.90, 30.40, 32.39, 33.10, 34.02, 34.18, 36.82, 39.24, 41.95, 43.63, 46.38, 46.88, 47.05, 47.44, 52.30, 52.98, 55.40, 121.23, 124.94 (C-12), 144.91 (C-13), 174.87 (C-28), 217.73 (C-3); m/z 551. [M + H]+; Anal. Calcd for C_36_H_58_N_2_O: C 78.49; H 10.61; N 5.09. Found: C 78.12; H, 10.54; N 4.89. 

*N*-(3-oxo-ursan-12-en-28-oyl)-ethylpiperazine **4**. Yield: 0.42 g (91%); m.p. 105 °C; [α]*_D_^20^* + 95 (c 0.05, CHCl_3_); ^1^H NMR (CDCl_3_, δ, ppm): 0.76, 0.82, 0.84, 0.91, 1.00, 1.00, 1.04, 1.04 (24H, 8s, 8CH_3_), 1.06–2.20 (23H, m, CH, CH_2_), 2.28–2.53 (6H, m, 3CH_2_), 3.53–3.71 (4H, m, 2CH_2_), 5.20 (1H, br.s, H-12); ^13^C NMR (CDCl_3_, δ, ppm): 11.75, 15.16, 16.90, 17.41,18.16, 19.58, 21.26 (2C), 21.48 (2C), 22.68, 23.27, 26.40, 28.19, 30.51, 32.59, 34.18, 34.25, 36.80, 38.70, 39.23, 39.37, 42.24, 45.22, 46.86, 47.40, 48.49, 52.30 (2C), 52.83 (2C), 55.31, 124.93 (C-12), 138.74 (C-13), 175.15 (C-28), 217.82 (C-3); m/z 551. [M + H]+; Anal. Calcd for C_36_H_58_N_2_O_2_: C 78.49; H 10.61; N 5.09. Found: C 78.30; H, 10.41; N 4.91.

#### 3.1.3. Synthesis of Compounds **5**–**10**

To a solution of compound **3** or **4** (1 mmol, 0.46 g) in ethanol (5 mL) under stirring, 2-pyridinecarboxaldehyde (0.14 g, 1.3 mmol), 3-pyridinecarboxaldehyde (0.14 g, 1.3 mmol) or furfural (0.13 g, 1.3 mmol), and 40% KOH in ethanol (2.5 mL) were added. The mixture was stirred for 24 h at room temperature, pH was adjusted to neutral by adding an aqueous solution of 5% HCl, and the mixture was poured into cold water (50 mL). The residue was filtered off, washed with water, and dried, then purified by column chromatography on Al_2_O_3_ with petroleum ether—CHCl_3_ (2:1 to 1:3) as eluent.

*N*-(2-{2-Pyridinylidene}-3-oxoolean-12-en-28-oyl)-ethylpiperazine **5**. Yield: 0.45 g (97%); m.p. 127 °C; [α]*_D_^20^* + 287 (c 0.05, CHCl_3_); ^1^H NMR (CDCl_3_, δ, ppm): 0.76, 0.82, 0.86, 0.87, 0.89, 1.08, 1.13, 1.19 (24H, 8s, 8CH_3_), 1.13–2.29 (22H, m, CH, CH_2_), 2.34–2.49 (2H, br.s, CH2), 2.86–3.10 (4H, m, 2CH_2_), 3.48–3.70 (4H, m, 2CH_2_), 5.23 (1H, t, J = 3.30, H-12), 7.05–7.70 (4H, m, H-1′, CH_arom_), 8.64 (1H, m, CH_arom_); 13C NMR (CDCl_3_, δ, ppm): 11.80, 15.41, 15.57, 16.48, 17.47, 18.31, 20.41, 21.27, 22.51, 22.65, 23.26, 23.57, 24.05, 27.26, 27.89, 28.18, 29.64, 29.68, 30.52, 33.07, 34.24, 36.01, 38.73, 38.94, 42.12, 44.56, 45.25, 46.41, 47.44, 52.28, 52.84, 52.93, 55.27, 121.45, 122.23, 122.26 (C-12), 136.04 (C-13), 144.82, 149.49, 155.52, 174.86 (C-28), 208.48 (C-3); m/z 640. [M + H]+; Anal. Calcd for C_42_H_61_N_3_O_2_: C 78.83; H 9.61; N 6.57. Found: C 78.45; H, 9.32; N 6.43.

*N*-(2-{3-Pyridinylidene}-3-oxoolean-12-en-28-oyl-ethylpiperazine **6**. Yield: 0.43 g (94%); m.p. 150 °C; [α]*_D_^20^* + 14 (c 0.05, CHCl_3_); ^1^H NMR (CDCl_3_, δ, ppm): 0.78, 0.84, 0.89, 0.91, 1.10, 1.13, 1.16, 1.47 (24H, 8s, 8CH_3_), 1.25–2.18 (20H, m, CH, CH_2_), 2.21 (1H, d, J = 16.0, H-1a), 2.40 (2H, br.s, CH_2_), 2.90–3.20 (4H, m, 2CH_2_), 3.50–73 (4H, m, 2CH_2_), 5.29 (1H, t, J = 3.31, H-12), 7.30–7.41 (2H, m, H-1′, CH_arom_), 7.70 (1H, d, J = 7.90, CH_arom_), 8.50–8.72 (2H, m, 2CH_arom_); ^13^C NMR (CDCl3, δ, ppm): 11.62, 15.29, 15.43, 16.50, 17.42, 18.32, 20.35, 21.26, 22.62, 23.61, 24.05, 25.78, 27.87, 28.16, 29.66, 30.41, 32.01, 33.06, 34.02, 36.36, 38.78, 39.01, 42.13, 43.74, 44.15, 45.24, 45.52, 46.38, 47.47, 52.33, 52.85, 53.02, 55.27, 121.11, 123.27, 125.23 (C-12), 136.74 (C-13), 145.00, 149.98, 151.37, 174.90 (C-28), 207.48 (C-3); m/z 640. [M + H]+; Anal. Calcd for C_42_H_61_N_3_O_2_: C 78.83; H 9.61; N 6.57. Found: C 78.54; H, 9.43; N 6.31.

*N-*(2-{Furfurylidene}-3-oxo-olean-12-en-28-oyl)-ethylpiperazine **7**. Yield: 0.44 g (95%); m.p. 150 °C; [α]*_D_^20^* + 68 (c 0.05, CHCl_3_); ^1^H NMR (CDCl_3_, δ, ppm): 0.75, 0.82, 0.87, 1.00, 1.02, 1.06, 1.09, 1.12 (24H, 8s, 8CH_3_), 1.13–2.18 (20H, m, CH, CH_2_), 2.25–2.48 (2H, m, CH_2_), 2.88–3.10 (4H, m, 2CH_2_), 3.48–3.70 (4H, m, 2CH_2_), 5.24 (1H, s, CH, H-12), 6.40–7.50 (5H, m, H-1′, 4CH_arom_); ^13^C NMR (CDCl_3_, δ, ppm): 11.82, 15.62, 15.69, 16.43, 17.50, 20.42, 21.33, 22.44, 22.56, 23.61, 24.07, 25.73, 27.87, 28.20, 29.86, 30.36, 32.17, 33.06, 34.01, 35.72, 38.72, 39.08, 42.08, 44.76, 45.53, 46.43, 47.62, 52.23, 52.75, 52.86, 52.86, 112.30, 115.41, 121.41, 124.22 (C-12), 128.30, 131.0, 144.41 (C-13), 152.62, 174.76 (C-28), 207.00 (C-3); m/z 629. [M + H]+; Anal. Calcd for C_41_H_60_N_2_O_3_: C 78.30; H 9.62; N 4.45. Found: C 78.05; H 9.40; N 4.22.

*N*-(2-{2-Pyridinylidene}-3-oxoursan-12-en-28-oyl)-methylpiperazine **8**. Yield: 0.43 g (93%); m.p. 120 °C; [α]*_D_^20^* + 128 (c 0.05, CHCl_3_); ^1^H NMR (CDCl_3_, δ, ppm): 0.78, 0.86, 0.91, 0.96, 1.12, 1.18, 1.18, 1.19, (24H, 8s, 8CH_3_), 1.20–2.18 (20H, m, CH, CH_2_), 2.44 (1H, d, J = 17.0, H-1a), 2.50–2.62 (2H, m, CH_2_), 3.08–3.52 (4H, m, 2CH_2_), 3.52–3.76 (4H, m, 2CH_2_), 5.34 (1H, t, J = 3.20, H-12), 7.16–7.67 (5H, m, H-1′, 4CH_arom_); ^13^C NMR (CDCl_3_, δ, ppm): 11.29, 15.41 (2C), 16.50, 20.43, 22.53 (2C), 23.67, 24.06, 25.78, 27.94, 29.69, 30.00, 30.41, 32.06, 33.05, 34.03, 36.07, 39.02, 42.16, 43.78, 44.58, 45.28, 45.36, 46.22, 47.54, 52.34 (2C), 53.68, 53.00, 121.65, 122.21, 126.77 (C-12), 128.33, 134.34, 136.03, 138.25, 144.68 (C-13), 149.51, 155.61, 175.03 (C-28), 208.70 (C-3); m/z 640. [M + H]+; Anal. Calcd for C_42_H_61_N_3_O_2_: C 78.83; H 9.61; N 6.57. Found: C 78.58; H, 9.40; N 6.39. 

*N*-(2-{3-Pyridinylidene}-3-oxoursan-12-en-28-oyl)-ethylpiperazine **9**. Yield: 0.42g (91%); m.p. 173 °C; [α]*_D_^20^* + 43 (c 0.05, CHCl_3_); ^1^H NMR (CDCl_3_, δ, ppm): 0.76, 0.85, 0.89, 0.92, 1.10, 1.13, 1.16, 1.47 (24H, 8s, 8CH_3_), 1.20–2.25 (20H, m, CH, CH_2_), 2.28 (1H, d, J = 15.9, H-1a), 2.40–2.56 (2H, m, CH_2_), 2.90–3.10 (4H, m, 2CH_2_), 3.59–3.65 (4H, m, 2CH_2_), 5.29 (1H, t, J = 3.22, H-12), 7.24–7.42 (2H, m, H-1′, CH_arom_), 7.69 (1H, d, J = 8.0, CH_arom_), 8.52 (1H, d, J = 7.7, CH_arom_), 8.66 (1H, s, CH_arom_); ^13^C NMR (CDCl_3_, δ, ppm): 11.60, 15.29, 15.46, 16.50, 20.35, 22.60, 23.61, 24.06, 25.77, 27.89, 29.65, 29.91, 30.40, 32.02, 33.05, 34.03, 36.38, 39.03, 42.13, 43.76, 44.16, 45.04, 45.24, 45.55, 46.40, 47.49, 52.31, 52.85, 53.08, 121.13, 123.24 (C-12), 128.32, 131.78, 133.46, 135.92, 136.06, 136.7, 144.99 (C-13), 149.08, 151.35, 174.90 (C-28), 207.43 (C-3); m/z 640. [M + H]+; Anal. Calcd for C_42_H_61_N_3_O_2_: C 78.83; H 9.61; N 6.57. Found: C 78.52; H 9.56; N 6.37.

*N*-(2-{Furfurylidene}-3-oxoursan-12-en-28-oyl)- ethylpiperazine **10**. Yield: 0.44 g (96%); m.p. 156 °C; [α]*_D_^20^* + 68 (c 0.05, CHCl_3_); ^1^H NMR (CDCl_3_, δ, ppm): 0.80, 0.88, 0.89, 0.93, 1.07, 1.09, 1.15, 1.18 (24 H, 8s, 8CH_3_), 1.23–1.78 (19H, m, CH, CH_2_), 2.15 (1H, d, J = 17.0, H-1b), 2.21 (1H, d, J = 17.0, H-1a), 2.45–2.50 (2H, m, CH_2_), 3.04–3.13 (4H, m, 2CH_2_), 3.55–3.77 (4H, m, 2CH_2_), 5.30 (1H, t, J = 3.4, H-12), 6.47 (1H, dd, J = 3.3, J = 1.6, CH_arom_), 6.55 (1H, d, J = 3.3, CH_arom_), 7.29 (1H, s, CH_arom_), 7.52 (1H, s, CH_arom_); ^13^C NMR (CDCl_3_, δ, ppm): 11.79, 15.29, 15.66, 16.48, 20.46, 22.46, 22.72, 23.66, 24.09, 25.77, 27.91, 29.91, 30.41, 32.04, 33.07, 34.04, 35.78, 39.00, 42.15, 43.81, 44.39, 44.84, 45.17, 45.58, 46.48, 47.49, 48.07, 52.31, 52.79, 52.95 (2C), 112.18, 115.39, 121.34, 124.11 (C-12), 130.99, 144.34 (C-13), 145.00, 152.62, 174.89 (C-28), 207.30 (C-3); m/z 629. [M + H]+; Anal. Calcd for C_41_H_60_N_2_O_3_: C 78.30; H 9.62; N 4.45. Found: C 78.14; H 9.55; N 4.31.

### 3.2. NCI-60 Screening

Compounds **3**–**12** were tested at one dose assay (10^−5^ M) toward a panel of approximately sixty cancer cell lines representing different cancer types: leukemia, melanoma, lung, colon, CNS, ovarian, renal, prostate, and breast cancers. Primary anticancer assays were performed according to the NCI protocol, as described elsewhere (see e.g., http://dtp.nci.nih.gov accessed on 1 November 2018) [50,51,52,53,54]. The compounds were added at a single concentration, and the cell cultures were incubated for 48 h. The end point determinations were made with a protein-binding dye, sulforhodamine B (SRB). The results for each compound are reported as the percent growth (GP %) of treated cells compared to untreated control cells (negative numbers indicate cell kill). Compounds with considerable activity against all tested human tumor cell lines (**4**, **6,** and **9**) were selected for the advanced assay against a panel of approximately sixty tumor cell lines at 10-fold dilutions of five concentrations [50,51,52,53,54]. The percentage of growth was evaluated spectrophotometrically versus controls not treated with test agents after 48 h exposure and using SRB protein assay to estimate cell viability or growth. Three antitumor activity dose-response parameters were calculated for each cell line. Furthermore, mean graph midpoints (MG_MID) were calculated for each of the parameters, giving an average activity parameter over all cell lines for the tested compound. For the MG_MID calculation, insensitive cell lines were included with the highest concentration tested.

### 3.3. Cell Culture

Human melanoma cell lines: A375, RPMI, and SK-MEL-28 were purchased from the American Type Culture Collection (ATCC). A375 were cultured in Dulbecco’s Modified Eagle’s Medium (DMEM, Sigma-Aldrich, St. Louis, MO, USA) high-glucose medium supplemented with 10% fetal bovine serum (FBS, Gibco, ThermoFisher Scientific, Waltham, MA, USA) and 1% penicillin/Strep, 10,000 IU/mL (Sigma-Aldrich), and RPMI and SK-MEL-28 were cultured in Eagle’s Minimum Essential Medium (EMEM, Sigma-Aldrich) high-glucose medium supplemented with 10% fetal bovine serum (FBS, Gibco, ThermoFisher Scientific) and 1% penicillin/Strep, 10,000 IU/mL (Sigma-Aldrich). The cells were incubated under standard temperature conditions of 37 °C and humidity containing 5% CO_2_.

### 3.4. Compound Stability in Cell Culture Media

The stability of the assessed compounds in the culture media was evaluated by thin layer chromatography (TLC), this representing a viable and easy to achieve technique for this specific endpoint [57,58]. Compounds **4** and **6** were dissolved in DMSO, and the solutions were successively diluted to a 5 μM final concentration in culture media (DMEM and EMEM) so that the final DMSO concentration did not exceed 0.5%. The solutions were incubated for 72 h, after which the medium was extracted in CHCl_3._ TLC using silica-coated aluminum plates (60 W F_254S,_ Merck KGaA, Darmstadt, Germany) was achieved from the extracted solutions compared to the non-incubated test compound solutions, using as mobile phase petroleum ether: CHCl_3_ (1:3). The plate was successively developed with I_2_ vapors, UV light, and bromocresol green to determine various additional spots corresponding to some decomposition byproducts.

### 3.5. DAPI Assay

The DAPI assay was performed on three human melanoma cell lines: A375, RPMI, and SK-MEL-28. The cells were cultured in 24-well plates at 2 × 10^5^ cells/well. Two samples (compounds **4** and **6**) of three different concentrations (0.1, 1, and 5 μM) and three concentrations of DMSO (0.1, 1, 5 μM) were used to stimulate the cells. The immunofluorescent staining technique was performed according to the protocol in [92]. Thus, after 24 h of stimulation, the cells were fixed by adding 4% paraformaldehyde and incubating at room temperature for one hour. After fixation, cell permeability was performed with 2% Triton X solution in phosphate-buffered saline (PBS). After 30 min of incubation at room temperature, blocking of permeabilization was performed with 0.01% Triton X solution and incubation at RT for one hour. DAPI staining was used in a concentration of 300 nM to visualize the nuclei. The cells were analyzed by fluorescence microscope at 40× magnification using CellSens V1.15 software (Olympus, Tokyo, Japan) and Image J software.

### 3.6. The Chorioallantoic Membrane Assay

The basic CAM assay protocol makes use of fertilized chicken eggs, incubated at 37 °C, under controlled humidity. On the third day of incubation (embryonic day of development, EDD), a small opening was made at one end of the eggs and 5–6 mL of albumen were removed, thus facilitating the separation of the chorioallantoic membrane. On EDD 4, a window was cut on the upper side of the egg and subsequently resealed, returning the specimens to the incubator. On EDD 7 (0 h), 10 µL of each test solution were administered inside a plastic ring previously applied on the intensely vascularized surface of the CAM [93]. All compounds were tested at a concentration of 1 µM in DMSO 0.5%, which also represented the control sample. All samples were daily applied in the same volume, for 72 h. For each analyzed sample, 5 eggs were used, and all samples were applied in triplicate. Macroscopic evaluation was daily performed in ovo by means of stereomicroscopy (ZEISS SteREO Discovery.V8, Göttingen, Germany), and all images were registered and processed by Axiocam 105 color, AxioVision SE64. Rel. 4.9.1 Software, (ZEISS Göttingen, Germany), ImageJ (ImageJ Version 1.50e, https://imagej.nih.gov/ij/index.html, accessed on 3 June 2021) and GIMP software (GIMP v 2.8, https://www.gimp.org/, accessed on 3 June 2021).

### 3.7. HET-CAM Assay

The in vivo Hen’s Egg Chorioallantoic Membrane Test (HET-CAM) assay determines a potential irritant effect on the vascular plexus of the chorioallantoic membrane [94]. The HET-CAM method was carried out following ICCVAM recommendations published in November 2016 in Appendix G and adapted to our conditions [88,95,96]. Thus, a volume of 300 µL of control or test solution was applied and the modifications produced at the CAM level were monitored by means of stereomicroscopy (Discovery 8 Stereomicroscope, Zeiss), registering significant images (Axio CAM 105 color, Zeiss), before application after 5 min of contact with the samples. All images were processed using Zeiss ZEN software, Gimp 2.8, and ImageJ software.

Negative control was represented by distilled water and solvent control DMSO 0.5%, and positive control by sodium lauryl sulphate (SLS) 0.5% in distillate water. The test substances were tested in concentrations of 1 µM.

The observation time of the produced reactions was 5 min (300 s), and the time at which the occurrence of a particular reaction took place was noted in seconds. Finally, the following reactions were: hemorrhage—H (blood vessel bleeding), vascular lysis—L (disintegration of blood vessels), coagulation—C (intra- or extra-vascular protein denaturizing). A variety of analysis methods may be used to assess irritancy potential of test substances. One analysis method that has been used extensively is an irritation score (IS). The formula used to generate an IS value is:

(1) $$\mathrm{IS}=5\times \frac{301-\mathrm{Sec}\;H}{300}+7\times \frac{300-\mathrm{Sec}\;L}{300}+9\times \frac{301-\mathrm{Sec}\;C}{300}$$

where: H = hemorrhage; L = vessel lysis; C = coagulation; hemorrhage time (Sec H) = onset of hemorrhage reactions on CAM (in seconds); lysis time (Sec L) = onset of vessel lysis on CAM (in seconds); coagulation time (Sec C) = onset of coagulation formation on CAM (in seconds). Means values are obtained. The formula comprises a factor indicating the impact on vascular damage of the observed effect, for example, coagulation has the highest impact expressed by the multiplication factor 9. The IS values range on a scale between 0 and 21.

### 3.8. Molecular Docking

The current molecular docking method used in this study was previously reported [97,98]. Briefly, protein target structures were retrieved from the RCSB Protein Data Bank [99] (Table 7). Protein structures were optimized using Autodock Tools v1.5.6 (The Scripps Research Institute, La Jolla, CA, USA). Water molecules, the co-crystalized ligand, and unnecessary protein chains were removed from the protein structure file. Subsequently, Gesteiger charges were added to the protein. The target files were saved as the required pdbqt file format. The 2D structures of compounds **4** and **6** were converted into 3D structure files (uff force field) using PyRx’s embedded Open Babel function. For our current molecular docking protocol, we used PyRx v0.8 (The Scripps Research Institute, La Jolla, CA, USA), with Autodock Vina’s embedded scoring function [100]. The docking method was validated as previously described [97]. The search space grid box was defined in terms of coordinates and size (Table 7) to best fit the active binding site of the native ligand. Docking scores were recorded as ΔG binding energy values (kcal/mol). Ligand–protein binding interactions and graphical representation were achieved using Accelerys Discovery Studio 4.1 (Dassault Systems Biovia, San Diego, CA, USA).

### 3.9. Gene Expression Analysis by qRT-PCR

Total RNA was extracted using peqGold RNAPureTM Package (Peqlab Biotechnology GmbH, Erlangen, Germany) according to the manufacturer’s instructions, and a DS-11 spectrophotometer (DeNovix, Wilmington, DE, USA) was used to measure total concentration of RNA. Reverse transcription was performed using the Maxima^®^ First Strand cDNA Synthesis Kit (Fermentas, St. Leon-Rot, Germany), and the mix was incubated in the Tadvanced biometra product line (Analytik Jena AG, Jena, Germany) with a thermal program of 25 °C for 10 min, 85 °C for 5 min. Quantitative real-time PCR was conducted in a Quant Studio 5 real-time PCR system, (Thermo Fisher Scientific, Inc., Waltham, MA, USA), with analysis being performed in 20 µL reactions containing Power SYBR-Green PCR Master Mix (Thermo Fisher Scientific, Inc.). Cycling conditions were: 95 °C for 10 s followed by 40 cycles of denaturing at 95 °C for 15 s, and annealing and extension at 60 °C for 1 min. The primer pairs used are displayed in Table 8.

## 4. Conclusions

The present study reports the anticancer biological assessment of a series of novel synthesized ursonic and oleanonic acids’ derivatives. These compounds were screened against the NCI-60 cancer cell line panel. Compounds **4** and **6** emerged as the overall highest cytotoxic active tested agents and were further evaluated in order to identify an anti-apoptotic and anti-angiogenic targeted anticancer mechanism of action. By means of DAPI staining, it was determined that the tested compounds affected overall cell and nuclei morphology. The observed changes were consistent with induced apoptotic cell death. Both compounds also induced up-regulation of pro-apoptotic Bak and down-regulation of Bcl-XL and Bcl-2 anti-apoptotic genes, as shown by rtPCR analysis. In silico molecular docking calculations showed that compounds **4** and **6** exhibited high scores for Bcl-XL inhibition, suggesting that the structures may trigger programed cell death by directly targeting anti-apoptotic proteins, as well. Ex vivo determinations revealed that both compounds do not promote angiogenesis impairment, but at the same time, they also do not show a high irritation potential. Based on these findings, we may conclude that oleanonic and ursonic acid derivatives such as compound **4** and **6** are promising cytotoxic anticancer agents that, from a mechanistic perspective, mainly regulate various apoptosis-related cellular processes.

## Figures and Tables

**Table 1:** Anticancer screening data in concentration 10^−5^ M for compounds **3**–**10**.

Compound (NSC)	60 Cell Lines Assay in 1 Dose 10 µM Concentration					
	Mean Growth, %	Range of Growth, %	Most Sensitive Cell Lines	Growth % of the Most Sensitive Cell Lines	Positive Cytostatic Effect ^a^	Positive Cytotoxic Effect ^b^
**3 (D-811947/1)**	46.01	−35.82 to 80.36	HL-60(TB) (Leukemia)	−35.82	5/59	7/59
**4 (D-811946/1)**	−10.47	−85.67 to −41.54	MALME-3M (Melanoma) LOXINVI (Melanoma)	−85.67 −85.17	60/60	60/60
**5 (D-818127/1)**	86.73	49.25 to 115.49	CAKI-1 (Renal cancer)	49.25	0/59	0/59
**6 (D-818136/1)**	23.36	−91.03 to 83.75	SK-MEL-5	−91.03	30/60	4/60
			U251 (CNS cancer)	−88.15		
**7 (D-818138/1)**	78.38	16.53 to 109.58	HL-60(TB) (Leukemia)	16.53	1/59	1/59
**8 (D-8181948/1)**	92.36	74.76 to 107.55	LOXIMVI (Melanoma)	74.76	0/59	0/59
**9 (D-811949/1)**	25.33	−38.20 to 65.71	HL-60 (Luekemia)	−38.20	33/59	5/59
**10 (D-811951/1)**	68.42	31.17 to 106.80	SR (Leukemia)	31.17	2/59	2/59
			PC-3 (CNS Cancer)	32.08		

^a^ Ratio between number of cell lines with percent growth from 0 to 50 and total number of cell lines. ^b^ Ratio between number of cell lines with percent growth of <0 and total number of cell lines.

**Table 2:** Influence of compounds **4**, **6,** and **9** and standard drugs doxorubicin (DRB) and 5-fluorouracil (5-FU) on the growth of individual tumor cell panel.

Panel/Cell Line	Compound 4			Compound 6			Compound 9			DRB	5-FU
	GI_50_ ^a^, µM	TGI ^b^, µM	LC_50_^c^, µM	GI_50_ ^a^, µM	TGI ^b^, µM	LC_50_ ^c^, µM	GI_50_ ^a^, µM	TGI ^b^, µM	LC_50_ ^c^, µM	GI_50_ ^a^, µM	GI_50_ ^a^, µM
*Leukemia*											
CCRF-CEM											
HL-60(TB)	0.976	2.74	>50.0	2.10	9.02	>100	2.78	8.97	>100	0.08	9.97
K-562	0.980	2.18	4.84	0.443	3.34	>100	2.08	5.00	>100	0.19	2.30
MOLT-4	0.994	2.90	>50.0	1.43	>100	>100	2.63	7.44	>100	NT	3.58
RPMI-8226	0.809	1.99	4.90	1.03	5.20	>100	2.45	7.38	>100	0.03	0.35
SR	-	-	-	0.959	5.13	>100	2.18	6.35	>100	0.08	0.04
MG_MID ^d^	-	-	-	0.706	5.10	>100	2.38	7.09	>100	0.03	NT
*NSC lung cancer*											
A549/ATCC	1.47	5.98	21.8	2.62	13.4	>100	3.15	16.8	>100	0.06	0.18
EKVX	0.916	2.42	8.95	1.90	6.48	43.5	2.17	7.32	61.7	0.41	NT
HOP-62	1.38	3.09	>50.0	1.88	3.75	7.47	2.43	5.86	>100	0.07	0.39
HOP-92	0.713	2.06	8.22	1.06	3.26	10.1	1.57	5.73	38.8	0.10	77.9
NCI-H226	1.78	7.01	28.7	1.76	3.80	-	1.89	4.07	8.78	0.05	54.7
NCI-H23	1.08	2.90	11.6	2.10	5.85	27.8	2.13	5.91	31.8	0.15	0.33
NCI-H322M	1.57	5.43	16.8	2.87	9.25	33.9	2.28	8.27	28.8	NT	NT
NCI-H460	0.913	1.75	3.27	1.39	2.93	6.20	1.78	3.45	6.69	0.02	0.05
NCI-H522	0.831	1.75	3.69	1.61	3.75	8.73	1.66	3.86	-	0.03	7.27
MG_MID	1.18	3.60	17.00	1.91	5.83	29.71	2.12	6.81	47.07	0.11	20.12
*Colon Cancer*											
COLO 205	1.02	2.37	14.5	1.89	4.74	>100	2.19	5.12	>100	0.18	0.15
HCC-2998	0.922	1.82	3.60	1.58	3.12	6.17	1.55	3.00	5.79	0.26	0.05
HCT-116	0.798	1.57	3.09	1.01	2.22	4.89	1.69	3.55	7.43	0.08	0.22
HCT-15	0.977	2.39	7.41	1.08	2.71	6.79	1.66	3.26	6.41	6.46	0.11
HT29	0.913	2.05	4.59	1.71	4.67	44.5	1.93	5.36	>100	0.12	0.17
KM12	1.03	2.22	4.79	1.58	3.26	6.75	1.75	3.56	7.23	0.27	0.21
SW-620	0.902	1.76	3.44	1.78	3.88	8.46	1.90	4.14	9.05	0.09	0.92
MG_MID	0.94	2.03	5.92	1.52	3.51	25.37	1.81	4.00	33.70	1.06	0.26
*CNS cancer*											
SF-268	1.77	6.49	24.6	1.59	4.79	85.7	1.77	4.02	9.15	0.10	1.62
SF-295	0.820	1.59	3.08	1.66	3.15	5.97	1.63	3.05	5.70	0.10	NT
SF-539	0.843	1.54	2.80	1.62	3.00	5.55	1.66	3.05	5.58	0.12	0.06
SNB-19	0.849	1.72	3.47	1.68	3.46	7.12	1.79	3.74	7.82	0.04	3.81
SNB-75	1.03	3.82	17.2	-	-	-	1.57	8.91	39.9	0.07	78.7
U251	0.854	1.85	4.00	1.58	3.11	6.13	1.82	3.67	-	0.04	0.92
MG_MID	1.028	2.84	8.69	1.63	3.50	22.09	1.71	4.41	13.63	0.08	17.02
*Melanoma*											
LOX IMVI	0.821	1.52	2.80	1.36	2.73	5.45	1.58	3.06	5.91	0.07	0.24
MALME-3M	0.807	1.51	8.84	1.53	2.91	5.54	1.40	2.75	5.39	0.12	0.05
M14	0.794	1.61	3.28	1.60	3.15	6.20	-	-	-	0.18	0.98
MDA-MB-435	1.05	2.30	5.22	1.48	3.11	6.52	1.70	3.49	7.18	0.25	0.07
SK-MEL-2	1.29	3.33	16.2	1.93	3.96	8.16	2.12	4.76	65.5	0.17	56.7
SK-MEL-28	0.889	1.70	3.27	1.48	2.83	5.41	1.74	3.15	5.69	0.21	1.03
SK-MEL-5	0.888	1.60	2.89	1.63	3.00	5.54	1.75	3.14	5.63	0.08	0.46
UACC-257	0.985	2.19	4.88	1.61	4.29	33.8	2.21	5.95	>100	0.14	3.55
UACC-62	0.835	1.61	3.09	1.28	2.83	6.23	1.51	2.96	5.80	0.12	0.52
MG_MID	0.93	1.93	4.94	1.54	3.21	9.20	1.56	3.66	25.14	0.15	7.07
*Ovarian cancer*											
IGROV1	1.04	4.85	19.1	1.91	4.16	9.10	1.89	3.87	7.94	0.17	1.22
OVCAR-3	1.10	2.44	6.52	1.51	2.95	5.73	1.65	3.19	6.17	0.39	0.01
OVCAR-4	1.26	4.74	18.0	2.17	7.85	47.8	2.57	7.36	36.6	0.37	4.43
OVCAR-5	0.907	1.87	3.87	1.56	3.35	7.19	1.54	2.93	5.57	0.41	10.9
OVCAR-8	1.10	2.89	30.1	2.14	5.54	>100	2.32	6.29	>100	0.10	1.74
NCI/ADR-RES	1.08	3.24	18.3	1.57	3.46	7.60	1.59	3.31	6.91	7.16	0.31
SK-OV-3	1.15	4.83	17.2	4.19	1.68	76.1	2.70	9.02	90.1	0.22	21.8
MG_MID	1.19	3.55	16.16	2.15	4.14	36.22	2.04	5.16	36.18	1.26	5.77
*Ranal cancer*											
786-0	0.814	1.70	3.56	1.60	3.08	5.91	1.98	4.37	-	0.13	0.72
A498	0.927	2.42	7.92	1.66	3.53	7.48	1.90	4.05	8.65	0.10	0.35
ACHN	1.33	4.38	14.8	2.60	10.5	32.4	2.46	6.45	22.6	0.08	0.27
CAKI-1	0.946	2.96	11.2	2.08	11.8	45.3	2.12	11.8	41.9	0.95	0.07
RXF 393	0.822	1.74	3.69	1.42	2.77	5.41	1.45	2.83	5.53	0.10	2.61
SN12C	0.776	1.48	2.81	1.22	2.54	5.30	1.61	3.01	5.66	0.07	0.49
TK-10	1.27	4.14	20.3	3.86	15.4	54.1	2.87	9.40	59.3	NT	1.12
UO-31	0.726	1.42	2.79	1.35	2.67	5.32	1.36	2.65	5.19	0.49	1.42
MG_MID	0.95	2.53	8.38	1.97	6.54	20.15	1.97	5.57	21.26	0.27	0.88
*Prostate cancer*											
PC-3	0.875	3.17	18.3	1.00	2.79	7.78	1.49	3.85	9.92	0.32	2.36
DU-145	1.67	5.62	18.6	1.83	3.88	8.23	2.08	4.21	8.53	0.11	0.36
MG_MID	1.27	4.40	18.45	1.42	3.34	8.01	1.79	5.96	9.23	0.21	1.36
*Breast cancer*											
MCF7	0.892	2.03	4.63	1.40	3.07	6.71	1.64	3.42	7.14	0.03	0.07
MDA-MB-31/ATCC	0.779	1.50	2.88	1.40	2.84	5.78	1.75	3.59	7.39	0.51	6.60
HS 578T	1.07	2.68	26.5	1.83	5.37	>100	1.91	4.77	>100	0.33	9.77
BT-549	0.833	1.58	3.00	1.47	2.88	5.66	1.69	3.59	-	0.23	10.6
T-47D	1.33	3.89	>50.0	2.61	13.7	>100	2.79	13.4	>100	0.06	8.12
MDA-MB-468	0.931	2.32	9.27	1.28	3.94	19.7	1.65	3.43	7.1	0.05	NT
MG_MID	0.97	2.33	16.05	1.67	5.30	39.64	1.91	5.37	36.94	0.19	7.03
MG_MID_60_ ^e^	1.03	2.74	12.31	1.53	6.29	32.37	1.93	5.29	37.95	0.38	6.97

^a^ GI_50_ (molar concentration of the compound that inhibits 50% net cell growth), ^b^ TGI (molar concentration of the compound leading to the total inhibition), ^c^ LC_50_ (molar concentration of the compound leading to 50% net cell death, presented in negative logarithm), ^d^ MG_MID—mean graph midpoints, ^e^ MG_MID_60_—mean graph midpoints in the full panel.

**Table 3:** The selectivity indexes of compounds **4**, **6,** and **9** on the growth of tumor cell lines subpanel at the GI_50_, TGI, and LC_50_ levels.

Panel	Compound 4			Compound 6			Compound 9			DRB	5-FU
	SI (GI_50_)	SI (TGI)	SI (LC_50_)	SI (GI_50_)	SI (TGI)	SI (LC_50_)	SI (GI_50_)	SI (TGI)	SI (LC_50_)	SI (GI_50_)	SI (GI_50_)
*Leukemia*	1.10	1.12	0.45	1.38	0.30	0.32	0.80	0.75	0.38	4.75	2.14
*NSCL cancer*	0.87	0.76	0.72	0.80	1.08	1.09	0.91	0.78	0.81	3.45	0.35
*Colon Cancer*	1.10	1.35	2.08	1.01	1.99	1.28	1.07	1.32	1.13	0.36	26.81
*CNS cancer*	1.00	0.96	1.41	0.94	1.80	1.46	1.13	1.20	2.78	4.75	0.41
*Melanoma*	1.11	1.42	**2.49**	0.99	1.96	**3.52**	0.60	1.45	1.51	2.53	0.99
*Ovarian Cancer*	0.86	0.77	0.76	0.71	1.52	0.89	0.95	1.02	1.05	0.30	1.21
*Renal Cancer*	1.08	1.08	1.47	0.77	0.96	1.61	0.98	0.95	1.78	1.41	7.92
*Prostate cancer*	0.81	0.62	0.67	1.08	1.89	**4.04**	1.08	0.89	2.11	1.81	5.13
*Breast cancer*	1.06	1.17	0.77	0.92	1.17	0.82	1.01	0.98	1.03	2.00	0.99

Bold values represent best results.

**Table 4:** Expression of the BAK, Bax, BCL-2, and BCL-XL genes treated with 1 μM solutions of compounds **4** and **6** and DMSO. Three replicates were used for each determination. Data are shown on as mean with one standard deviation. Marked boxes (*) indicate significant differences as compared to the reference group (Student’s *t* test, *** *p* < 0.001, ** *p* < 0.01, * *p* < 0.05).

Sample	Bcl-XL		Bcl-2		BAK		BAX	
A375								
	Mean	Std.Dev	Mean	Std.Dev	Mean	Std.Dev	Mean	Std.Dev
DMSO 1	4.550	(2.694)	2.216	(0.485)	5.200	(0.085)	4.063	(0.644)
Compound **4**	0.500 *	(0.292)	0.7366 **	(0.133)	0.706 ***	(0.255)	0.270 ***	(0.069)
Compound **6**	0.300	(0.265)	1.166	(0.683)	0.516 ***	(0.231)	0.590 ***	(0.157)
				RPMI				
DMSO 1	18.563	(6.786)	4.063	(0.374)	4.163	(0.710)	3.040	(0.723)
Compound **4**	*ND*	*ND*	1.483 *	(1.165)	156.243 ***	(23.330)	0.460 **	(0.324)
Compound **6**	1.050 *	(0.900)	0.236 ***	(0.185)	11.853 **	(1.789)	0.640 **	(0.310)
SK-Mel-28								
DMSO 1	4.730	(0.353)	2.573	(1.015)	1.383	(0.249)	2.086	(0.322)
Compound **4**	0.086 ***	(0.066)	0.083 *	(0.056)	101.820 **	(37.304)	0.023 ***	(0.011)
Compound **6**	0.003 ***	(0.005)	0.083 **	(0.032)	91.283 ***	(14.461)	0.140 ***	(0.117)

*ND*—Not determined.

**Table 5:** Recorded docking scores for compounds **4** and **6** (binding energy, ∆G kcal/mol).

Target PDB ID	Binding Free Energy ∆G (kcal/mol)		
	Co-Crystalized Ligand	Compound 4	Compound 6
2W3L	−10.3	−6.5	−6.7
2YXJ	−10.7	−8.5	−9.2
3EQG	−8.8	−6.7	1.8
4FA6	−9.3	8.7	24.2
4JT5	−8.5	3.1	24.7
4OQ5	−12.2	−2.8	37.3

**Table 6:** The irritant potential of compounds **4** and **6**.

Test Compound and Controls	Irritation Score	Type of Effect
Distillate water	0	Non-irritant
SLS 0.5%	17.03	Strong irritant
DMSO 0.5%	0.68	Non-irritant
Compound 4	0	Non-irritant
Compound 6	0	Non-irritant

**Table 7:** Molecular docking parameters and protein targets.

PDB ID	Protein	Grid Box Size	Grid Box Center Coordinates	Conformers
2YXJ	Apoptosis regulator Bcl-X (Bcl-XL)	size_x = 18.8213065002 size_y = 30.6345174449 size_z = 10.2997599705	center_x = −10.2573467499 center_y = −18.1808412775 center_z = 9.46376920765	8
3EQG	Dual specificity mitogen-activated protein kinase kinase 1 (MEK1)	size_x = 14.6058170561 size_y = 16.2060321551 size_z = 8.70490192182	center_x = −4.16588471722 center_y = 59.3845284049 center_z = 34.6388482191	8
2W3L	Apoptosis regulator Bcl-2 (Bcl-2)	size_x = 15.4649240353 size_y = 13.2408048336 size_z = 13.3102000528	center_x = 37.1927426166 center_y = 26.8056607992 center_z = −12.8141402336	8
4OQ5	Induced myeloid leukemia cell differentiation protein (Mcl-1)	size_x = 15.5072934727 size_y = 13.9772309154 size_z = 10.4111215841	center_x = 11.6262580805 center_y = 3.65908070556 center_z = 7.90001930158	8
4JT5	Mammalian target of rapamycin-target of rapamycin complex subunit LST8 (mTOR-LST8)	size_x = 16.0737519631 size_y = 11.2048088799 size_z = 10.5072207821	center_x = 51.5677297505 center_y = −1.9363339261 center_z = −48.4627944976	8
4FA6	Phosphatidylinositol 4,5-bisphosphate 3-kinase catalytic subunit gamma isoform (PI3Kγ)	size_x = 14.3022779071 size_y = 10.3234871012 size_z = 8.72203337452	center_x = 44.8020362414 center_y = 13.746321532 center_z = 30.3837942752	8

**Table 8:** Primer pairs used in PCR analysis.

	Forward	Reverse
BAK	5′ ATGGTCACCTTACCTCTGCAA 3′	5′TCATAGCGTCGGTTGATGTCG 3′
BCL-XL	5′ GATCCCCATGGCAGCAGTAAAGCAAG 3′	5′CCCCATCCCGGAAGAGTTCATTCACT 3′
Bax	5′ GCCGGGTTGTCGCCCTTTT 3′	5′CCGCTCCCGGAGGAAGTCCA 3′
Bcl-2	5′-CGGGAGATGTCGCCCCTGGT-3′	5′-GCATGCTGGGGCCGTACAGT-3′

## Data Availability

The data presented in this study are available on request from the corresponding authors.

## Supplementary Materials

The following are available online at https://www.mdpi.com/article/10.3390/ijms222010967/s1.
